# Are self-report of disability pension and long-term sickness absence accurate? Comparisons of self-reported interview data with national register data in a Swedish twin cohort

**DOI:** 10.1186/1471-2458-10-763

**Published:** 2010-12-15

**Authors:** Pia Svedberg, Annina Ropponen, Paul Lichtenstein, Kristina Alexanderson

**Affiliations:** 1 Division of Insurance Medicine, Department of Clinical Neuroscience, Karolinska Institutet, Stockholm, Sweden; 2 Faculty of Medicine, Institute of Biomedicine, Ergonomics, University of Eastern Finland, Kuopio, Finland; 3 Department of Medical Epidemiology and Biostatistics, Karolinska Institutet, Stockholm, Sweden

## Abstract

**Background:**

Self-reported disability pension (DP) and sickness absence are commonly used in epidemiological and other studies as a measure of exposure or even as an outcome. The aims were (1) to compare such self-reports with national register information in order to evaluate the validity of self-reported DP and sickness absence, and (2) to estimate the concordance of reporting behaviour in different twin zygosity groups, also by sex.

**Methods:**

All Swedish twins born 1933-1958 who participated in the Screening Across the Lifespan Twin study (SALT) 1998-2003, were included (31,122 individuals). The self-reported DP and long-term sickness absence (LTSA) at the time of interview was compared to the corresponding register information retrieved from the National Social Insurance Agency by calculating the proportions of agreements, kappa, sensitivity, specificity, concordance rates, and chi-square test, to evaluate construct validity.

**Results:**

The proportions of overall agreement were 96% and specificity 99% for both DP and LTSA, while the sensitivity was 70% for DP and 45% for LTSA. Kappa estimates were 0.76 for DP, and 0.58 for LTSA. The proportions of positive agreement were 64% for DP and 42% for LTSA. No difference in response style was found between zygosity groups among complete twin pairs for DP and LTSA. Results were similar for women and men and across age. Kappa estimates for DP differed somewhat depending on years of education, 0.68 (college/university) vs. 0.77 (less than 13 years in school) but not for LTSA.

**Conclusions:**

Self-reported DP data may be very useful in studies when register information is not available, however, register data is preferred especially for LTSA. The same degree of twin similarity was found for truthful self-report of DP and LTSA in both monozygotic and dizygotic twin pairs. Thus, the response style was not influenced by genetic factors. One consequence of this would be that when estimating the relative importance of genetic and environmental effects from twin models, heritability estimates would not be biased.

## Background

Disability pension (DP) and sickness absence can be seen as proxies of reduced health or as social consequences of disease, and such work absences might cause severe problems for the individual, employer, and for the society [1,2]. During the past decade, research on DP and sickness absence has gained increased attention and studies have shown that both DP and long-term sickness absence (LTSA) are multifactor phenomena that are influenced by a broad variety of risk factors [3,4]. Nevertheless, decisions on sickness benefits are to be based on reduced work capacity on medical grounds. In epidemiological studies, self-reported data on DP and sickness absence are frequently used as exposure and/or outcome measures. However, misclassification related to the use of self-reported data might lead to considerable bias.

To date, there are relatively few studies that have investigated the validity of information from self-reported sickness absence [5-17] and even fewer of self-reported DP [11,18]. It is also still controversial as to what extent self-reported data are reliable and valid [10]. Proper conclusions from self-reported data on DP and sickness absence require accurate estimations and therefore the validity of such data needs to be investigated further [19].

Previous studies of comparisons between self-reported and register data from employers or insurance organisations have focused on a variety of different sickness-absence measures. Most studies have compared self-reported number of sickness-absence days during a specific time period (weekly, monthly, or yearly) compared to employer or administrative register data [7-9,11,16], others validated sickness-absence days due to specific diagnoses [10,12,15] as compared to register data. The two recent studies that investigated validity of self-reported DP showed good agreements between the two sources of information using national register data [11,18]. Concordances between self-reported and register information have also been estimated with somewhat different measure of agreements such as sensitivity, specificity, or kappa statistics which makes comparisons between studies somewhat difficult. In general though, studies of self-reported and register data of short-term sickness absence, with short recall periods, and DP have shown better agreements than comparisons of self-reports and register data on LTSA. Most previous studies investigated the validity of self-reports using retrospectively collected information on sickness absence, often short-term sickness absence, and in general, the studies having a shorter recall period showed better agreements between the sources of information [1]. Further, most studies have also been based on rather small samples (n ≤ 600), although a few recent studies are based on larger populations, such as the Whitehall II study in London (n = 8220) [13], the Swedish study HAKuL (n = 4900) [8], and the Norwegian study by Hartz and colleagues (n = 17,244) [18]. Moreover, several of the previous studies have been based on selected cohorts such as specific patient [11,12] or occupational groups [8,14].

Twin studies often also use self-reported data when assessing reason for individual differences in terms of genetic and environmental influences of a trait or disease. Thus, these studies also rely on that accurateness of self-reported data is not influenced by twin status (zygosity) in itself, i.e. whether the twin is identical (monozygotic (MZ)) or fraternal (dizygotic (DZ)), should not influence the way of reporting presence or absence of health symptoms, diseases, or other factors in surveys. To the best of our knowledge, there is no twin study of validity of self-reported DP or sickness absence to date. Other studies on validity of health phenomenon or diseases in twin settings have shown mixed results and few studies have been able to evaluate those unaffected. For example, validity of self-reported hyperthyroidism and hypothyroidism have shown to be unsatisfactorily low [20], while higher rates of agreements were found for osteoarthritis [21]. It is, therefore, also of interest to investigate the concordances between self-reporting behaviour of DP and LTSA with national register data among twin pairs. Comparisons between zygosity groups also provide information on whether genetic or environmental factors contribute to reporting behaviour. Genetic influences are indicated if the MZ twin pairs more often are concordant for reporting behaviour compared to DZ twin pairs [22], while equal concordances between zygosity groups indicate that it would be possible to exclude genetic influences on response style.

The aims were 1) to compare self-reported data with national register information in order to evaluate the construct validity of self-reported DP and LTSA, and 2) to study whether the correspondence between self-report and register data of DP and LTSA was equal for monozygotic (MZ) and dizygotic (DZ) twins, and across sex. Hence, the second aim was to assess the concordance of reporting behaviour for MZ and DZ twin pairs.

## Method

### Source population

The source population was twins in the Swedish Twin study of Disability pension and Sickness absence (STODS). This study includes all twins born in Sweden between 1925 and 1958, identified through the Swedish Twin Registry (STR) (29,799 complete twin pairs) [23,24]. Information available in STODS include twin administrative variables (zygosity, sex, pair status), data from two previously conducted studies by STR: the Screening Across the Life-span Twin (SALT) computer-assisted telephone-interview study, and a questionnaire in 1973 (Q73). Data about sickness absence and DP from the National Social Insurance Agency (MiDAS-database), socio-demography from Statistics Sweden, and mortality from the National Board of Health and Welfare, were linked to all individuals.

### Study sample

In this cross-sectional study the sample consist of the 31,122 twins (52% women) who participated and answered questions about their work situation, or absence from work, in the SALT-study. The interviews were conducted between January 1998 and March 2003 and the SALT-study has been described in more detail elsewhere [23,24]. At the time of interview, age ranged between 41 to 65 years (born 1933-1958), mean age was 54 years. The sample consist of 12,237 complete twin pairs (3247 MZ pairs, 4421 DZ pairs, 4461 opposite sexed DZ pairs, and 108 pairs with unknown zygosity), and 6648 twin individuals, i.e. their twin partner did not participate in the SALT-study. Hence, 31,122 twins were included in the agreement analyses of the whole sample, and 12,129 complete twin pairs with known zygosity were included in the pair analysis.

### Data sources and measures

From the SALT-study, the question about current work situation was phrased as follows: "Which one of the following alternatives describes your current situation best?" With response alternatives "Working 100%", "Working part-time", "Disability pensioned", "Long-term sickness absent", "Retired (old age)", "Maternity leave", "House wife/man", "Unemployed", "Student", "Self-employed/entrepreneur", or "Military service". The exact date of when the interview was conducted was logged in the computer system, enabling comparisons with the national records of DP and LTSA for that specific time point. Age, sex, education (< 13 years in school, ≥ 13 years in school (college/university)), and zygosity were also derived from STR and SALT [23,24].

Register information on having received benefits for DP and LTSA (start and ending dates of episodes) were linked to all twins using the unique ten digit Swedish identification number of each twin individual. The MiDAS-database includes all individuals living in Sweden that are older than 16 years of age and who have been granted DP, or who were sick listed for more than 14 uninterrupted days. Data about DP and LTSA in the MiDAS-database were available from 1993 onwards. All people living in Sweden who are between 16-64 year of age are covered by the national sickness insurance, covering up to 80% income lost due to work incapacity for medical reasons.

Two response categories were created from the self-reported data; yes/no responses to being granted DP at the time of interview, and yes/no responses to being on LTSA at the time of interview. Similarly two categories were created based on the MiDAS-database information on whether the individual had an ongoing sickness absence episode or DP at the time of interview or not (yes/no).

### Statistical analysis

The agreements were calculated, assuming national register data to be correct, using cross-tabulations and reported as proportions of overall agreement (P_o_), positive agreement (PA), and negative agreement (NA). *Sensitivity *was calculated as the number of true positive i.e. DP/LTSA individuals correctly identified as such divided by the total number of true positive plus those individuals on DP/LTSA but not identified as such, while *specificity *was calculated as individuals correctly classified as not being on DP or LTSA divided by the total number of these individuals without DP/sickness absence benefits plus those individuals who had no such benefit but incorrectly classified as being on DP/LTSA. *Kappa *(*k*) statistics was calculated for the binary responses on the questions of whether being on LTSA/DP or not. The *k *value reflects the agreement between observations adjusted for agreements occurring by chance. To define poor, slight, fair, moderate, substantial, and almost perfect agreement, arbitrary categories of *k *values (0.00, 0.00-0.20, 0.21-0.40, 0.41-0.60, 0.61-0.80, and 0.81-1.00, respectively) were used as suggested by Landis and Koch [25].

In order to analyze whether MZ and DZ twin pairs show the same degree of correspondence of self-reporting behavior and register information of DP and LTSA, we compared the proportions of MZ and DZ twin pairs, also by sex, that were discordant or concordant (i.e., where both twins self-report agreed with register data, or where both twins self-report did not agree with register data) for response behavior and Chi-square test statistics were used.

This project was approved by the Regional Ethical Review Board of Stockholm, Sweden (2007/524-31).

## Results

**Figure 1:**
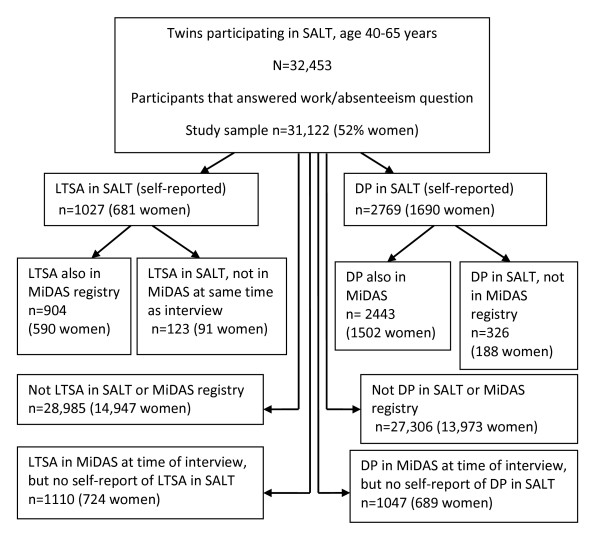
**Description of the study sample: occurrence of self-reported (SALT) and national register information (MiDAS) of disability pension (DP) and long-term sickness absence (LTSA) in the Swedish twin cohort**.

A description of the twin cohort and distribution of self-reports and register information on DP and LTSA is presented in Figure 1.

### Disability pension

In total 2769 individuals (1690 women, 1079 men) of the study sample (n = 31,122) indicated that they were on DP at the time of the interview (8.9%), and of these 2443 twins were also registered in MiDAS (88%). According to the MiDAS records, the prevalence of DP was 11.2% (n = 3490) and these individuals should have reported they were on DP at the time of interview. Results for DP show a high overall (P_o_) agreement, a high negative agreement (NA), specificity and a substantial Kappa estimate while sensitivity and positive agreement (PA) was somewhat lower (Table 1). Results were similar for women and men. Post hoc Kappa calculations across age groups and educational levels showed substantial agreements and no differences between age groups, *k *values for individuals < 55 years was *k *= 0.74 and for individuals ≥ 55 years *k *= 0.75. Results of DP agreement differed depending on years of education i.e. a higher *k *value was found for the group with less than 13 years in school (*k *= 0.77), than for the group with ≥ 13 years in school (i.e. college and university), *k *= 0.68.

**Table 1:** Measures of agreement between self-reported and national register data on disability pension (DP) and long-term sickness absence (LTSA) in a Swedish twin cohort (n = 31,122)

Measure of agreement	DP			LTSA		
	**All**	**Women**	**Men**	**All**	**Women**	**Men**

**P_o_**	96%	95%	97%	96%	95%	97%
**PA**	64%	63%	65%	42%	42%	43%
**NA**	95%	94%	96%	96%	95%	97%
**Sensitivity**	70%	68%	72%	45%	45%	45%
**Specificity**	99%	99%	99%	99%	99%	100%
**Kappa**	0.76	0.74	0.77	0.58	0.57	0.59

*Notes: *P_o _= Proportion overall agreement; PA = Proportion Positive agreement; NA = Proportion Negative agreement

### Sickness absence

In total 1027 individuals (681 women, 346 men) indicated they were on LTSA at the time of interview (3.3%), and of those, 904 were also registered having a sickness absence episode in MiDAS (88%). According to the national records (MiDAS), the prevalence of sickness absence was 6.5% (n = 2014), hence individuals that should have reported they were on LTSA at the time of interview. A majority of the twins who reported LTSA and who were also registered in MiDAS (902 of 904) were on full-time sickness absence, at least at some time during their sickness absence episode according to the records. Results for LTSA show a high overall agreement, negative agreement, specificity, and a moderate Kappa estimate while sensitivity and positive agreement was lower (Table 1). Results were similar for women and men. Post hoc Kappa calculations across age groups and educational levels showed moderate agreements and no differences between groups. Kappa values for individuals < 55 years was *k *= 0.59 and for individuals ≥ 55 years *k *= 0.56. Results across educational level were similar (less than 13 years in school, *k *= 0.58, and ≥ 13 years in school (i.e. college and university), *k *= 0.56).

### Twin pair correspondence

Concordance between register and self-reported data for DP and LTSA were equal between zygosity groups and across sex (Table 2). Chi-square test yield no statistically significant differences between zygosity groups for DP (p = 0.31) or LTSA (p = 0.64), and results were similar when stratified by sex. There were few pairs in which both members reported incorrectly being on LTSA or DP. In total, the number of concordant female twin pairs in which both members of the pair reported incorrectly being on LTSA as compared to the male pairs were 8/2 in MZ twins and 4/1 in DZ twins, and the number of concordant female twin pairs in which both members of the pair reported incorrectly being on DP as compared to the male pairs were 9/3 in MZ twins and 5/6 in DZ twins.

**Table 2:** Number of twin pairs (n) by zygosity and sex; reporting behaviour on agreement between self-report and register data of disability pension (DP) and long-term sickness absence (LTSA) in a Swedish twin cohort

		DP		LTSA	
**Groups**	**Twin pairs (n)**	**^a^Concordant pairs (n)**	**Discordant pairs (n)**	**^a^Concordant pairs (n)**	**Discordant pairs (n)**

**Monozygotic (MZ)**					
Female	1816	1642	174	1640	176
Male	1431	1355	76	1359	72
All MZ	3247	2997	250	2999	248
**Dizygotic (DZ)**					
Female same-sexed	2403	2159	244	2195	208
Male same-sexed	2018	1888	130	1913	105
All same-sexed	4421	4047	374	4108	313
Opposite sexed	4461	4115	346	4136	325
All DZ	8882	8162	720	8244	638

**Total**	12,129	11,159	970	11,243	886

**Note: ^a^**Concordant pairs include pairs where both members accurately reported that they were, or were not granted DP or LTSA benefits plus those pairs concordant for inaccurately reporting (non corresponding information between self-report and register data) about being on DP or LTSA benefits

## Discussion

The present study of a large Swedish twin cohort evaluated the agreement between self-reported DP and LTSA using interview data and national insurance register data in order to assess the validity of self-reported data. A substantial agreement was found between self-report and register data on DP, while there was only a moderate agreement between the two data sources of information for LTSA. Further, the correspondence between self-reporting behaviour and register data on DP and LTSA was found to be equal for MZ and DZ twin pairs and, thereby, in line with expectations of equal concordance between self-report and register information across zygosity groups. These results indicate that the response style was not influenced by genetic factors, rather by environmental factors.

High specificity and high proportion of negative agreement in the present study show that individuals not on DP or LTSA could be correctly classified based on self-reports, in line with previous findings [5,8,12,15]. The presence of underreporting of LTSA and DP might be due to unwillingness to reveal these personal circumstances of being supported by sickness-absence benefits. Such a behavior has been recognized for self-reports of other health indicators, when people tend to consider the information to be sensitive, such as smoking habits and weight. On the other hand, the SALT interview study covers many areas of health behavior, socioeconomic circumstances, as well as questions about common complex diseases, and it was therefore considered unlikely that questions about work/absence from work would have intimidated the participants.

Good agreements between self-reported and register data have previously been reported for DP in two Norwegian studies [11,18], and moderate to good agreements for sickness absence [5-10,12-16], in similar studies. However, most of these previous studies compared self-report with employers' administrative records of DP and sickness absence and not with national official insurance registries. Further, all but the two validity studies of DP [11,18] used retrospectively collected data with recall periods of number of sickness-absence spells or days that varied between a few weeks up to several years, hence, most probably, recall bias was introduced [19]. Level of agreement seems to be dependent on length of sickness absence and recall period, since previous findings show better agreements for short-term sickness absence and for shorter recall periods. The two studies on DP though [11,18] were able to compare self-report to register data at the same time, however, the exact dates of self-reports were not assessed in the questionnaires in these studies which still leave some uncertainty. The present study, on the other hand was not subject to this kind of recall bias due to different time spans since we were able to compare the specific date of the interview responses with the national registry of both DP and LTSA. Even though specificity was high, low sensitivity, i.e. to what extent self-reported data correspond to national register data as reference, and low proportion of positive agreement especially for LTSA, indicates that self-reported data on LTSA are to be used with caution as exposure or outcome measure. Previous studies have reported both similar and higher sensitivities ranging from 55 to 91% for DP and sickness absence [5-8,11,18]. In the present study, there were only minor sex differences in agreements between self-reported and register data on DP or LTSA, in contrast to some previous findings [8,9,11,13], of somewhat higher rates of agreements between the two sources of data for men. Post hoc kappa analyses for age and educational groups showed that agreements of LTSA and DP were equal across age. Kappa values for DP differed somewhat depending on education i.e. a higher agreement was found for those with less than 13 years in school. Previous results of agreements for sickness absence and DP from Norway point in the same direction, but differences between educational groups were minor [11].

Another concern with self-reported data is that of measurement error related to interpretation and comprehension by respondents [18,26,27]. In the present study, the response alternative "long-term sickness absent" might not have been the very best one, since "long-term" might be interpreted in many different ways by the respondents. For someone who is seldom sickness absent, one week might be considered long-term, while for someone else, a period of four weeks of sickness absence might not be considered long. It is, therefore, likely that level of agreement for LTSA in the present study was influenced by the individuals' interpretation and comprehension of the response alternative, especially since almost all respondents answering they were on LTSA were on full-time sickness absence at time of interview. Hence, the validity would be less influenced by the effect of grade of sickness absence, that is, being on full- or part time sickness absence-something that is possible in Sweden. The response alternative "disability pension" might have been interpreted more straightforward by the respondents even though underreporting of DP was recognized. In Sweden, DP benefits are generally granted first after a long period of sickness absence due to reduced work capacity on medical grounds, a decision that later on has been re-evaluated and become a more permanent benefit. Self-reported DP should therefore be less influenced by measurement error related to interpretation. Part-time DP benefits can also be granted in Sweden which might explain the underreporting of DP in this study and suggests that in countries with only full-time DP benefits, estimates of agreement might be higher.

Analysis of a large population based twin cohort enabled estimation of twin pairs correspondence of respondents' reporting of DP and LTSA, no such results have so far been presented to our knowledge. High degrees of concordances were found, equal between zygosity groups and across sex. The overall impression is, therefore, that these results imply that we can trust self-reports from both identical and fraternal twin pairs to be valid to the same degree. In addition, equal concordance for MZ and DZ twin groups suggests that reporting of DP and LTSA are not influenced by genetic factors, rather that environmental factors play a role in reporting behaviour [28,29]. Factors influencing self-reports overall, such as how questions are formulated and interview techniques, are most likely to be of great importance and hence related to the already discussed matter of interpretation and comprehension of questions and response alternatives by respondents. Based on these findings it is advised that surveys of sickness absence or DP should include validated instruments, or in absence of such, clearly phrased questions, that are not subject to a variety of interpretations in interviews or questionnaires [26,27]. However, preferable is having access to insurance register data.

A limitation in this study was the inability to validate self-reported short-term sickness absence, that alternative was not raised in the interview nor does the national register cover sickness-absence spells that are shorter than 14 days. Another limitation was that the response alternative "long-term sickness absent" in the survey did not specify what was meant by "long-term". Strengths were the large cohort from the population-based Swedish Twin Registry, national insurance register information of the National Insurance Agency on DP and LTSA of very high quality - the later is due to that it is not a research register but an national register of all monetary payments for these types of benefits, no drop-out, i.e., all individuals participating in the SALT interview were included in the national insurance register. The current study has no recall bias and, thus, may have a better validity than previous reports that used several months recall periods. Also, by studying twins it was possible to exclude genetic influences on response style.

## Conclusions

Self-reported data on DP and LTSA may be useful in studies when register data are not at hand, however, results point in favour of using register information especially for LTSA. Use of self-reported data could also lead to underestimation of the prevalence of DP and LTSA in a population. The twin analyses showed the same degree of twin similarity for truthful self-report of DP and LTSA in both MZ and DZ twins. Thus, the response style does not seem to be influenced by genetic factors [28,29], which is congruent with the interpretation that how questions are formulated and interview techniques (i.e., measurement error) rather than response style are of importance. One consequence of this would be that when estimating the relative importance of genetic and environmental effects from twin models, heritability estimates would not be biased. Clearly, it is advised that surveys of sickness absence or DP should include validated instruments, or in absence of such, clearly phrased questions, that are not subject to a variety of interpretations in interviews or questionnaires.

## Competing interests

The authors declare that they have no competing interests.

## Authors' contributions

PS was a principal investigator on the study and designed the study, collected and analysed data, wrote the first draft of the paper and lead the critical review and revision of the manuscript. AR, PL and KA all contributed substantially to the study design, interpretation of results, drafting and review of the manuscript. All authors have approved the final version of the manuscript for publication.

## Pre-publication history

The pre-publication history for this paper can be accessed here:

http://www.biomedcentral.com/1471-2458/10/763/prepub
